# Comparison of the effectiveness of different ırrigation activation methods on biofilm removal in lateral canals – an ın vitro study

**DOI:** 10.2340/aos.v84.43737

**Published:** 2025-05-20

**Authors:** Uğur Aydın, Bilal Turan, Emre Çulha, Muazzez Naz Baştürk Özer, Melih Özdemir

**Affiliations:** Department of Endodontics, Gaziantep University, Gaziantep, Turkey

**Keywords:** Biofilm mimicking, EndoActivator, hydrogel, self-adjusting file, ultrasonic system

## Abstract

**Objectives:**

Activation of sodium hypochlorite (NaOCl) to remove biofilm from lateral canal is important for the success of endodontic treatment. This study aimed to compare the biofilm dissolving capacity of different irrigation techniques in resin blocks with two lateral canals manufactured with 3D printers.

**Materials and methods:**

Biofilm-mimicking hydrogel was placed in the upper and lower lateral canals of 75 resin blocks. Main canals of the blocks were irrigated with 5% NaOCl. Then, the blocks were randomly divided into five groups: sonic activation (SA), passive ultrasonic irrigation (PUI), intracanal heating (ICH), self-adjusting file (SAF) and control. The amount of hydrogel removed was measured by weighing the blocks before and after the treatment and further visually scored. Data were analyzed using Kruskall Wallis H, Wilcoxon, Tukey Post-hoc HSD (Honestly Significant Difference) and one-way ANOVA tests.

**Results:**

The SAF group showed the highest hydrogel scores compared to other groups (*p* < 0.05). The hydrogel dissolution capacity of the SA group was lower compared to SAF, PUI and ICH techniques (*p* < 0.05). Based on visual scoring, SAF group was superior to other activation methods (*p* < 0.05) which are similar to each other (*p* > 0.05) in both upper and lower lateral canals.

**Conclusion:**

All activation methods were superior than the control group. The SAF system demonstrated superior hydrogel dissolving ability, while SA, PUI, and ICH groups showed similar effectiveness.

## Introduction

The purpose of irrigation in root canal treatment is to increase the cleansing and disinfection of the root canal system. Periapical lesions can be prevented by eliminating all pathogens from the root canal system [1]. Eradicating the layer of biofilm that along root canal walls via mechanical preparation alone is not possible. [2]. One of the leading causes of this insufficiency is structural abnormalities in the root canals, such as lateral canals that are inaccessible during instrumentation and might contain bacterial biofilm [3]. The disinfection procedure during chemomechanical preparation, which irrigates the deeper parts of the root canal system with antibacterial solutions, is critical to the effectiveness of endodontic treatment [4]. In the contaminated root canal area, bacteria can either adhere to the dentin or to other microorganisms to create a biofilm, or they may shift around as a planktonic unit. In the contaminated root canal area, bacteria can either adhere to the dentin or to other microorganisms to create a biofilm. Furthermore they may shift around as a planktonic unit [5, 6]. The bacteria within the biofilm exhibit various phenotypes and unique features, than the same microorganisms in planktonic form. One of these distinctive characteristics is the enhanced capacity for resisting antimicrobial medications that a species in a developed biofilm has, which can be 100–1,000 times higher than that of the same species developed planktonically [7]. Planktonic microorganisms are less challenging to remove from the root canal than bacteria in biofilm form. Other bacteria, on the other hand, attach to walls and quickly build a biofilm that has viscoelastic qualities, which allow for feeding, and protect against chemical and mechanical damage [8, 9].

Sodium hypochlorite (NaOCl) is frequently utilized for root canal irrigation because of its broad antibacterial spectrum and tissue dissolving function [10, 11]. NaOCl, however, has to come into close interaction with biofilms in order to demonstrate its efficacy. Under clinical circumstances, undegradated and remaining biofilm tend to be typical after full-strength NaOCl treatment [3]. In order to make NaOCl possible to reach the deeper and narrower parts in the root canal system, a variety of irrigation activation techniques have been designed including manual dynamic activation, sonic activation (SA), passive ultrasonic irrigation (PUI), and laser activation [12–14]. PUI and SA are two widely used activation methods for irrigation solutions during final irrigation [15]. The key difference between them is the frequency spectrum in which they are utilized. In the course of PUI, a file oscillates at 25–30 kHz in a pattern of motion that includes nodes and anti-nodes [16]. The file, on the other hand, oscillates at frequencies ranging from 1 to 6 kHz when the irrigant is agitated with SA [17]. Using different frequency ranges of PUI dramatically reduces the number of microorganisms in the root canal system following chemomechanical preparation. PUI devices make possible irrigant penetration, which leads to biofilm elimination from lateral canals when compared to SA [18]. The EndoActivator is an SA device with three types of flexible polymer tips and a cordless handpiece. This device increases the effectiveness of irrigation compared to traditional needle irrigation [19].

Self-adjusting file (SAF) is a hollow, compressible NiTi file without a central metal core. During the mechanical preparation, the SAF system’s hollow reciprocating tool enables concurrent irrigation. The company (ReDent Nova, Raanana, Israel) states that the SAF may three-dimensionally adjust to the geometry of the root canal [20]. The uniform stripping of dentin is facilitated by the abrasive surface of the lattice filaments when SAF is used in an in-and-out motion [21]. SAF application to large oval root canals was found to be more successful than syringe irrigation in decreasing *Enterococcus faecalis* levels [22].

The intracanal heating (ICH) technique, which uses heat carrier tips, is an alternative strategy for activating NaOCl in root canals [23]. Higher free available chlorine concentrations from heated NaOCl lead to better collagen breakdown. When compared to pre-heated NaOCl irrigation, ICH of NaOCl cleans noticeably more debris from the root canal system [24].

Previous investigations on the impact of irrigation processes on biofilm employed extracted human teeth or gypsum converted to hydroxyapatite, which makes it difficult to see the removal of biofilm-mimicking materials directly [25, 26]. Three-dimensional (3D) printing resin models, on the other hand, give transparency and the ability to generate numerous specimens with the same anatomical characteristics, allowing researchers to explore the dynamic relationship between activated NaOCl and biofilm removal during irrigation. Additionally, viscoelastic materials like hydrogels in endodontic resin models may simulate the viscoelastic features of biofilm [27].

The purpose of this study is to assess the effects of SA, PUI, ICH and SAF techniques on eliminating biofilm in the lateral canals utilizing the hydrogel biofilm mimicking method on resin models manufactured on a 3-D printer. The null hypothesis was that there would be no significant differences regarding the irrigation procedures removed hydrogel from the lateral canals.

## Materials and methods

### Determining sample size

Power calculation was achieved using G*Power 3.9.1 software (Heinrich Heine University, Dusseldorf, Germany) to determine whether the expectation of a medium effect size (*f* = 0.25) between measurements was statistically significant. The minimum number required in each category was 15 (α= 0.05; 1−β = 0.80).

### Preparation of the root canal models

**Figure 1:**
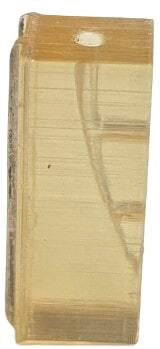
Resin block manufactured from PMMA with two lateral canals.

75 polymethylmethacrylate (PMMA)-based root canal models were created using a 3D printer (MakerBot, Brooklyn, USA), with one main canal and two lateral canals. The main root canal was 20 mm long and tapered to 30/0.04. The lateral canals were created between 7 and 14 mm coronal from the apical, with dimensions of 5 and 3 mm length, 0.5 mm orifice wide, and no taper. This unique design enhanced the efficacy of biofilm removal to be clearly monitored in both the lateral and major root canals (Figure 1).

### Forming the hydrogel biofilm

The hydrogel was produced using 3 g gelatin (Merck, New Jersey, USA) and 0.06 g hyaluronan (95% sodium hyaluronate; Fisher, Waltham, MA, USA). All of these ingredients were dissolved in 45 mL of distilled water at 50°C. To observe the biofilm, 0.25 g of red food coloring (KTC, Wednesbury, UK) was added to the combination. This hydrogel material was examined in a pilot research using thermocouples, which revealed that it solidified after 1 min in a model at 21°C room temperature. As a result, the hydrogel biofilm material was kept in liquid form in a water bath at 30°C prior to the usage.

**Figure 2:**
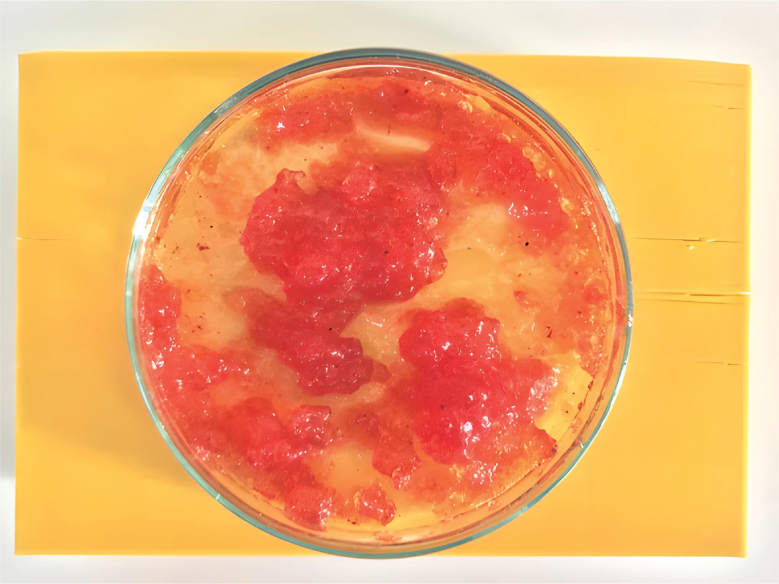
Hydrogel produced to mimic biofilm.

The produced hydrogel biofilm structure (Figure 2) was applied to the model’s lateral canals with hand tools. The resin models were then randomly categorized into five groups (SAF, SA, PUI, ICH, and control), each including 15 models, using an online randomization tool (Randomizer, https://www.randomizer.org/). Each root canal model was irrigated with 20 ml of 5% NaOCl solution (Microvem, Istanbul, Turkey) for a total of 2 min before the procedures. A 27-gauge endodontic irrigation needle (DiaDent, Seoul, South Korea) was used for irrigation; however, it remained 2 mm shorter than the length of the root canal.

Control group: Artificial canals of the control group were irrigated with 20 ml of 5% sodium hypochlorite for 2 min by using irrigation needles as mentioned above. No further irrigation or activation was carried out.

**Figure 3:**
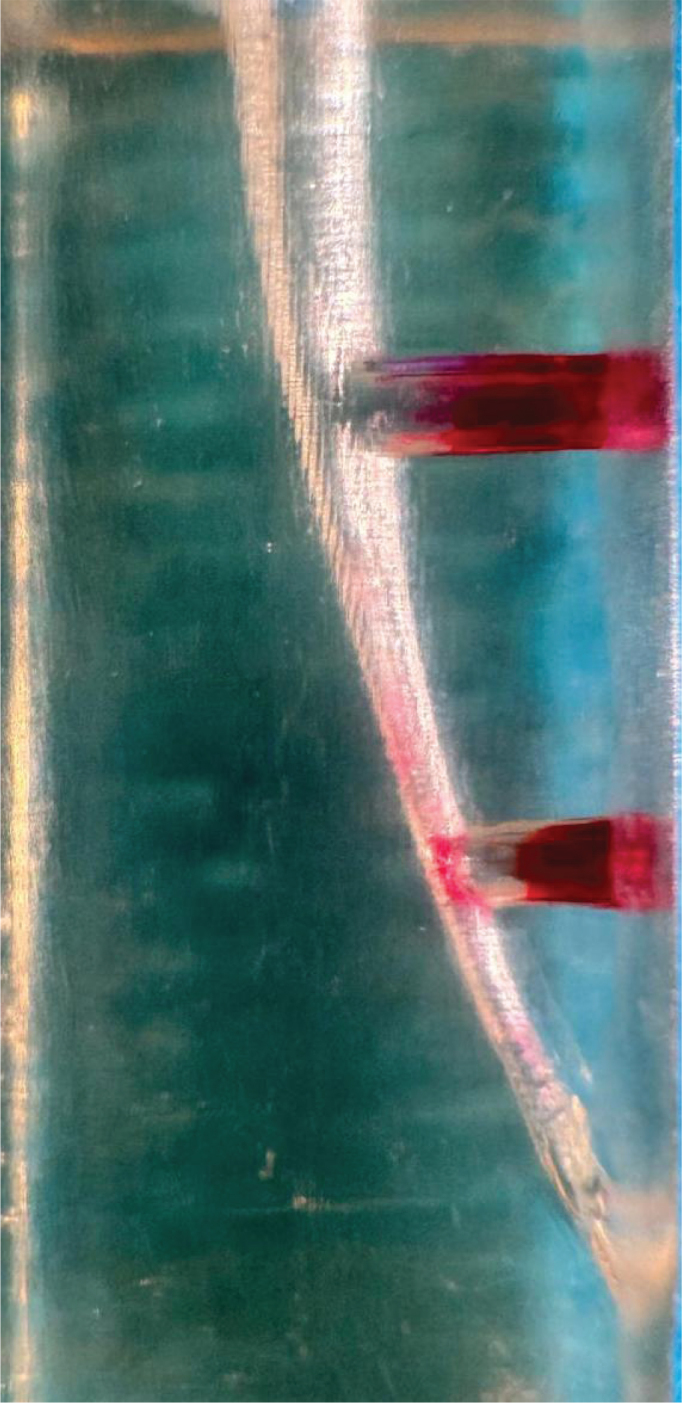
Image of the sample after SA.

SA group: The EndoActivator device (Dentsply-Sirona, Charlotte, NC, USA) operated for 30 s with the 25.04 tip positioned 3 mm shorter than the canal length. This procedure was carried out four times, while the root canal models included 5% NaOCl. For each model, an entirely new tip was used. The image of the post-experiment sample was taken (Figure 3).

**Figure 4:**
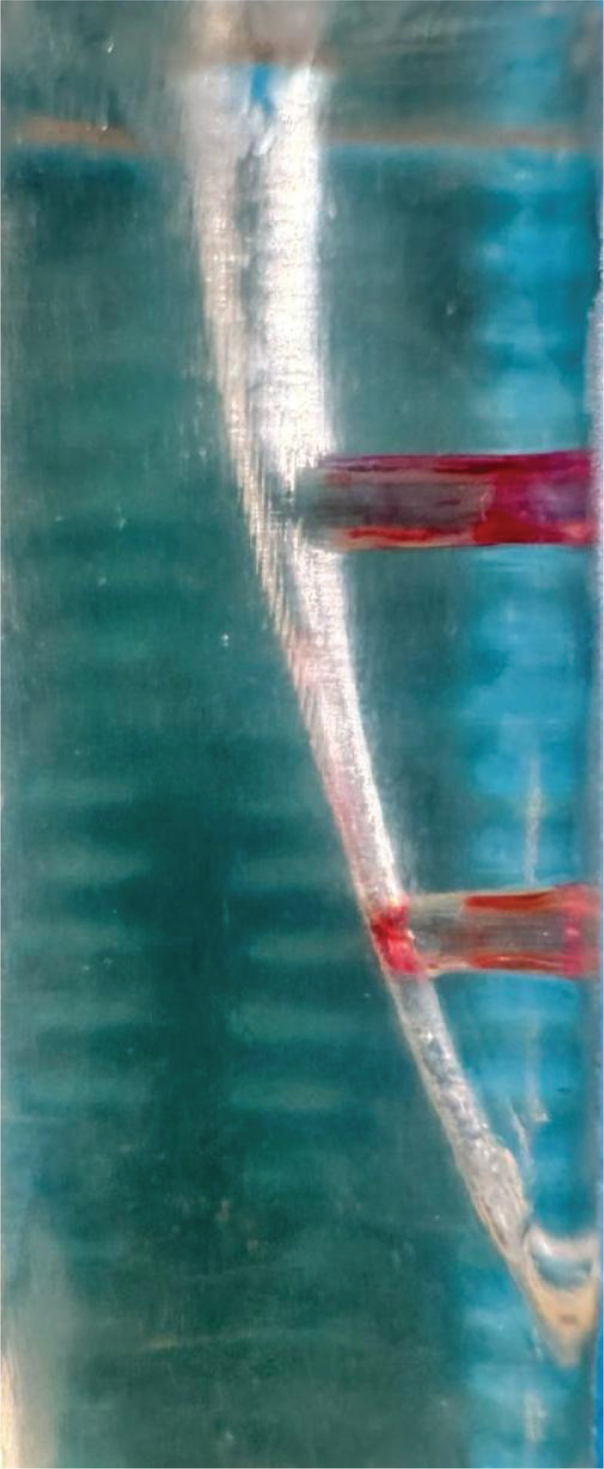
Image of the sample after PUI.

PUI group: While the resin models filled with 5% NaOCl, the Minipiezon ultrasonic irrigation system (EMS, Nyon, Switzerland) activated the E1 (0.7 mm) ultrasonic tip (Woodpecker, Guilin, China) for 15 s, positioning it 2 mm shorter than the canal length and using a power setting of 6 as instructed. The frequency employed under the mentioned conditions was approximately 30 kHz and 50–80 µm oscillation with 1 mm up and down motion based on previous studies [28, 29]. For each model, this procedure was performed four times, while the canals were filled with NaOCl. Every tip was tested in between procedures and applied to a maximum of five models. Image of the post experiment sample was taken (Figure 4).

**Figure 5:**
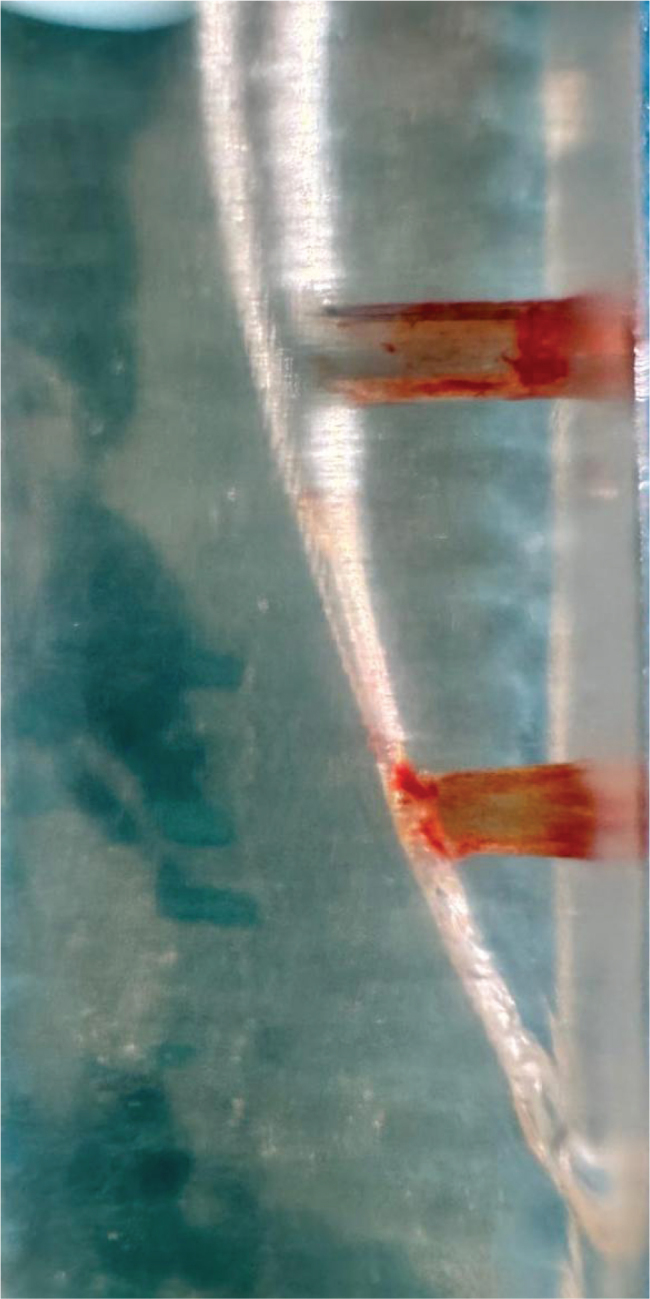
Image of the sample after ICH.

ICH group: ICH was carried out as performed by Jaiswal et al. [30]. In the presence of 5% NaOCl in the canal, a 40/0.025 heating tip from Fast-pack (Eighteeth Medical, Chang-Zhou, China) was administered for 5 s at 120°C. This process was carried out four times in the presence of NaOCl. Image of the post experiment sample was taken (Figure 5).

**Figure 6:**
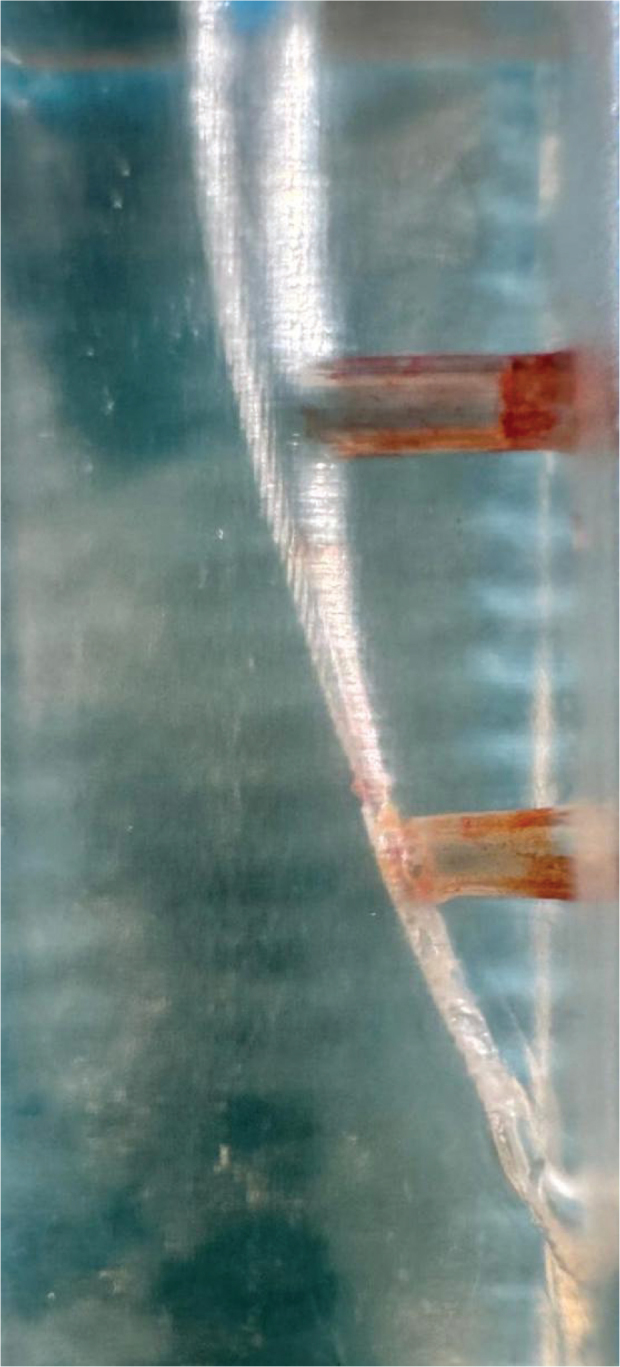
Image of the sample after SAF.

SAF group: The RDT3 head was equipped with a Kavo Gentle power low-speed handpiece (Kaltenbach and Voigt, Biberach, Germany). Utilizing a VATEA peristaltic pump (ReDent-Nova, Ra’anana, Israel), irrigation was carried out for 2 min with a #1 SAF file (ReDent-Nova, Ra’anana, Israel) and 20 ml of 5% NaOCl delivered to the canals at a flow rate of 10 ml/min. Three models were prepared with each SAF tip. Image of the post experiment sample was taken (Figure 6).

### Measurement after irrigation procedure

Following various irrigation techniques, paper points were used to dry the canals (Pearl Endo, Gyonggi-Do, South Korea). To get rid of moisture in the canals, all models were left at room temperature for 2 h. In order to calculate the amount of removed hydrogel, each root canal model was weighed before and after application using a 10^-5^ precision weight measuring instrument (Shimadzu, Kyoto, Japan). These results were recorded individually for each root canal model. The quantity of change in dissolved hydrogel was measured in grams. Furthermore, the volume of dissolved hydrogel in the gaps of the lower and upper lateral canals connected to the main canal was visually scored using a stereomicroscope (Carl Zeiss, Gottingen, Germany) at 4x magnification in every phase (Figure 3). Artificial illumination was provided by an LED light source (LA-HDF7010; Hayashi, Tokyo, Japan).

The scoring index used to visualize the remaining hydrogel in the lateral canals of the resin models in the study is as follows:

0: No dissolution occurred.

1: Dissolution occurred in approximately 1/3 of the lateral canal.

2: Dissolved in about half of the lateral canal

3: Dissolution occurred in at least 2/3 of the lateral canal.

All observations and weight measurements were made separately by two clinicians with previous laboratory work experience. The averages of these measurements were used for statistical analysis. According to the scoring system, higher scores meant more hydrogel removal from the lateral canals.

### Statistical analysis

IBM SPSS V23 was utilized for analyzing the data (Chicago, USA). The Shapiro Wilk test was performed to determine if the data represented normal distribution. Data which were not normally distributed were analyzed by using Kruskall Wallis H test. The weight changes among the groups were compared before and after the procedure using the Wilcoxon, Tukey Post-hoc HSD and one-way ANOVA (Analysis of Variance) tests. The median was used to display the analysis results for the quantitative data. The significance level was determined as *p* < 0.05.

## Results

Table 1 represents weight changes and standard deviation of the groups. All study groups were statistically superior to the control group (*p* < 0.05). The SAF group removed significantly more biofilm than the others (*p* < 0.05). The PUI, SA and ICH groups removed statistically similar degree of biofilm (*p* > 0.05).

**Table 1:** Weight differences of root canal models before and after the procedure according to groups (g).

Groups	*N*	Mean weight	Standard deviation
Sonic activation	15	0.01432**^b^**	0.00610
Passive ultrasonic irrigation	15	0.01824**^b^**	0.00476
Intracanal heating	15	0.01617**^b^**	0.00584
Self-adjusting file	15	0.02356**^a^**	0.00567
Control	15	0.00694**^c^**	0.003355

* Different superscript lowercase letters indicate statistical difference among the groups.

Considering the scoring system that evaluates hydrogel removal, the SAF group had a higher mean for hydrogel removal from both upper and lower lateral canals compared to other groups (*p* < 0.05). The PUI, SA and ICH groups were statistically similar to each other (*p* > 0.05) and superior than the control group (*p* < 0.05) (Tables 2 and 3).

**Table 2:** Mean and standard deviation analysis of hydrogel scoring of the upper lateral canals.

Groups	*N*	Mean	Standard deviation
Sonic activation	15	1.46666^b^ (0.74581 ± 7.52285)	0.46972
Passive ultrasonic irrigation	15	1.73333^b^ (0.89525 ± 8.23318)	0.47213
Intracanal heating	15	1.66666^b^ (0.73269 ± 7.98565)	0.57213
Self-adjusting file	15	2.46666^a^ (1.68741 ± 12.25475)	0.62716
Control	15	0.6667^c^	0.46972

* Different superscript lowercase letters indicate statistical difference among the groups.

**Table 3:** Mean and standard deviation analysis of hydrogel scoring of the lower lateral canals.

Groups	*N*	Mean	Standard deviation
Sonic activation	15	0.6666^b^ (0.2356 ± 3.9540)	0.52666
Passive ultrasonic irrigation	15	1.4343^b^ (0.9525 ± 8.6328)	0.47766
Intracanal heating	15	1.2224^b^ (0.7185 ± 7.1229)	0.50888
Self-adjusting file	15	1.73333^a^ (1.1247 ± 9.2556)	0.69999
Control	15	0^c^	0

* Different superscript lowercase letters indicate statistical difference among the groups.

## Discussion

In the present study, hydrogel was utilized to replicate the biofilm in the lateral canals because it exhibits viscoelastic characteristics comparable to other biofilm models, has standard properties, and is convenient for usage [27]. However, there are differences between natural biofilms and synthetic hydrogel. Firstly, natural biofilms comprise substances that are not found in hydrogel, which can hinder the biofilm removal efficiency of irrigation activation strategies. SSecondly, natural biofilms includes water canals that influence the biofilm’s adhesive capacity [31]. Finally, hydrogel’s attachment to the resin block surface is difficult to maintain, and it develops a single layer over the surface. In contrast, natural biofilms attach aggressively to surfaces in layers [32]. Despite these differences in physical properties, hydrogel is the most preferred material to mimic biofilm.

In our study, each of the active irrigation groups (SA, PUI, ICH, SAF) decreased the quantity of hydrogel in the resin models when compared to the control group. Previous studies found that there is no significant difference among syringe irrigation and activation methods including sonic and/or ultrasonic activation in terms of root canal disinfection and particularly biofilm removal in the main root canal [33, 34]. However, other studies stated that syringe irrigation alone cannot provide enough irrigant penetration towards lateral canals [35–37]. These findings may explain why all activation methods removed more hydrogel compared to syringe irrigation.

Another finding of the present study is that the SAF system removed more hydrogel from both the upper and lower lateral canals than the other activation methods. Thus, the null hyphothesis is rejected. The situation might be related to the simultaneous delivery of NaOCl throughout the apertures of the braided metal cage structure of SAF tips with instrumentation. The special design of the SAF files allows the fit of the file to the canal morphology and by this way its abrasive strips contact with all dentin walls [21]. During filing motion, the flow of NaOCl which is forced towards canal walls may enhance its penetration to lateral canals. This instrumentation pattern of SAF leaves less unprepared regions in root canals than rotary file systems. As mentioned above, all activation methods increased the penetration of NaOCl to lateral canals. However, this unique mechanism of action of the SAF system seems to provide further irrigant penetration to lateral canals both coronally and apically compared to other techniques. The potential benefits of SAF in decreasing biofilm in difficult-to-reach areas of apical anatomical structures have been documented in the literature. According to Siqueira et al. SAF was more effective in reducing *E. faecalis* populations from root canal systems than rotary titanium instrumentation [22]. Besides, SAF, further widening the main canal of the resin model by eroding it, may have allowed NaOCl to have better access to the lateral canals and led to more effective removal of the hydrogel.

Ultrasonic activation is the most popular activation method and is considered as a gold standard, compared with other techniques. Ultrasonic tips vibrate at 25–30 kHz, generate acoustic streamings and increase the penetration of irrigants towards lateral canals. Cavitation effect forming bubbles following acoustic streaming increases shear stresses [38]. The oscillating pressure created by the ultrasonic tip is transmitted to the hydrogel, causing the hydrogel’s connection to become weaker [39]. When a certain threshold is reached, some of the hydrogel separates [40]. Furthermore, during PUI, the temperature in the root canal system increases as a result of conversion of kinetic energy to heat which may have an impact on NaOCl’s ability to remove biofilms [24]. It should be noted that for free oscillation and improved effect of ultrasonic tips, adequate canal preparation, which was reported as at least 30–35 by Retsas and Boutsioukis [41], should be performed. The present study protocole included blocks with 30/04 main canals. However, according to our results, PUI resulted in partial dissolution of hydrogel structure which is also true for other activation methods. The dissolution of a viscoelastic substance from lateral canals via ultrasonic irrigation activation is difficult to optimize because viscoelastic hydrogel does not have a consistent removal process. While the oscillatory stream factor is particularly strong at the ultrasonic tip, it diminishes quickly as it goes away. According to previous studies, NaOCl reduces the efficiency in all irrigation groups 1 mm inward from the lateral canal orifice and its impact diminishes beyond 3 mm [42, 43]. This may be the result of decreased volume of NaOCl being exchanged and transported. In addition, only the top layers of the biofilm encounter functional NaOCl diffusion [44], and free available Cl ions are quickly absorbed during the interaction between NaOCl and the biofilm [45]. Furthermore, it is obvious that hydrogel removal is more in upper lateral canals for all groups which is due to the reduced jet stream and vapor lock effect in the apical region. Of course, the inability of the included methods to totally remove biofilm from lateral canals may also be linked to the placement of huge amounts of hydrogel in artificial lateral canals.

Both weight differences and scoring of upper/lower lateral canals were similar between SA and PUI groups. Unlike ultrasonic activation systems, sonic activation handpieces are equipped with plastic tips and operate at lower frequencies (1–6 kHz). In the present study, EndoActivator system was used for sonic activation. However, its oscillation frequency is 160–190 Hz, which is much lower than the defined range for sonic systems. In the study of Jiang et al. [46], it was stated that EndoActivator device is less effective than ultrasonic system which is related to the relatively higher oscillation amplitude of EndoActivator (1200 µm for EndoActivator, 50–80 µm for ultrasonic devices), resulting in unintentional wall contact avoiding irrigant streaming and cavitation effects. In the present study, artificial lateral canals with a diameter of 0.3 mm (300 µm) were included. Studies using a scanning electron microscope reported that the lateral canal diameters in human teeth have a width between 10 and 200 µm [35, 47]. However, 10–200 µm width range was not sufficient for accurate visualization of hydrogel. This realtively wide opening compared to natural lateral canal orifice may ease the penetration of irrigant even activated with low frequency. This may have resulted in similar finding in our study which may be different in favor of ultrasonic systems in natural root canal morphology.

Based on the findings of the present study, ICH of NaOCl increased the removal of hydrogel from lateral canals similar to PUI and SA. According to a previous study [48], heating NaOCl up to 60°C increases its tissue dissolving and organic remnant removal capacity. In a more novel approach, increasing the heat of NaOCl inside the root canals up to 180°C provides a further increase in biofilm removal [49]. This situation results from the fact that heating NaOCl accelerates ions and expedites the chemical reaction between NaOCl and biofilm [50]. On the inner surface of the main canal wall and the lateral canal, ICH treatment also raises the temperature; however, the heat impact diminishes with increasing distance from the canal center. The efficiency of eliminating the biofilm layer in the lateral canals declines in parallel with the reduction in temperature caused by ICH [23]. ICH is more successful in clearing remaining organic tissues and hard tissue debris from the main root canal zone than preheated and unheated NaOCl [49]. In addition, during the ICH applied during our study, the lower lateral canal in the resin model was exposed to less temperature than the upper one. This may have caused the ICH activation used in our study to be more effective in the upper lateral canals, where the heated tip was closer.

To exclude factors like dentin composition or root canal morphology, all activation tasks were carried out with the same canal length, shape, apical diameter, taper, and lateral canal dimensions. Similar to previous studies, the transparent PMMA material of examined models permitted direct observation of lateral canals following irrigation without the need to modify the model – a step that might have an impact on the intracanal conditions [51, 52]. The selection of transparent resin-based 3D printed models enabled direct viewing of hydrogel elimination using various irrigation techniques [53]. Furthermore, an exact representation of the anatomy of a basic root canal was provided [54]. Additionally, the hydrogel was positioned precisely in each sample’s upper and lower lateral canals. In order to regulate the hydrogel’s viscoelastic characteristics, which are crucial to the stability of the hydrogel, temperature conditions in the laboratory were also observed [27]. However, the highly standardized setting of the present study may not accurately reflect clinical conditions due to its experimental nature. The lateral canal structure in natural teeth is significantly smaller than the lateral canal we utilized in our study. Therefore, the activation methods may have shown a better effect in these large lateral canals. This is a limitation of our study. On the other hand, it is known that activation methods increase penetration into the lateral canals [12]. Although our study does not simulate the clinical scenario, it has value in terms of comparing activation methods.

## Conclusion

All irrigation techniques increased the biofilm removal capacity of NaOCl. The SAF system was more effective than other groups. SA, ICH, and PUI had similar effect in removing the biofilm-mimicking hydrogel in the lateral canals. More research is needed to determine how these findings can be applied to clinical practice.

## Data Availability

No datasets were generated or analyzed during the current study.
